# Antidepressant pharmacological mechanisms: focusing on the regulation of autophagy

**DOI:** 10.3389/fphar.2023.1287234

**Published:** 2023-11-09

**Authors:** Shimeng Lv, Guangheng Zhang, Yufei Huang, Jiamin Li, Ni Yang, Yitong Lu, Haoteng Ma, Yuexiang Ma, Jing Teng

**Affiliations:** ^1^ Department of First Clinical Medical College, Shandong University of Traditional Chinese Medicine, Jinan, China; ^2^ Ruijin Hospital Affiliated to Shanghai Jiaotong University School of Medicine, Shanghai, China; ^3^ College of Traditional Chinese Medicine, Shandong University of Traditional Chinese Medicine, Jinan, China

**Keywords:** depression, autophagy, Traditional Chinese medicine, pharmacological mechanisms, antidepressant

## Abstract

The core symptoms of depression are anhedonia and persistent hopelessness. Selective serotonin reuptake inhibitors (SSRIs) and their related medications are commonly used for clinical treatment, despite their significant adverse effects. Traditional Chinese medicine with its multiple targets, channels, and compounds, exhibit immense potential in treating depression. Autophagy, a vital process in depression pathology, has emerged as a promising target for intervention. This review summarized the pharmacological mechanisms of antidepressants by regulating autophagy. We presented insights from recent studies, discussed current research limitations, and proposed new strategies for basic research and their clinical application in depression.

## 1 Introduction

Major depressive disorder (MDD), also known as depression, is a mood disorder characterized by enduring feelings of sadness and anhedonia. It is a significant contributor to global suicide rates. According to the World Health Organization, depression affects approximately 4.4% of the global population, exceeding 350 million individuals. By 2030, depression is projected to become the leading cause of global burden of disease and non-fatal health-related losses (Rehm and Shield, 2019; Bayes et al., 2020). Treatment of depression primarily involves the use of selective serotonin reuptake inhibitors (SSRIs) and related medications. SSRIs exert their pharmacological action by selectively inhibiting serotonin (5-HT) transporters, prolonging and enhancing the effects of 5-HT, thereby exhibiting antidepressant properties (Perez-Caballero et al., 2014; Bi et al., 2022). However, SSRIs have adverse reactions such as nausea, headache, chronic sexual dysfunction, and weight gain (Wang et al., 2019a). Furthermore, they often have delayed onset and high non-response rates (Qu et al., 2021; Wei et al., 2022). Therefore, there is a need for safer and more effective antidepressants. Traditional Chinese medicine (TCM) offers promise due to its diverse components, targets, and modes of action. Moreover, it has been demonstrated that the active components and compounds found in TCM have shown notable effectiveness in treating depression with minimal side effects (Chi et al., 2019). Consequently, TCM has become a prominent area of scientific investigation for the management of depressive disorders.

Autophagy is a crucial intracellular degradation mechanism where cellular components are transported and broken down in lysosomes. Additionally, autophagy functions as a dynamic circulatory system that generates fresh molecular constituents and energy to maintain cellular renewal and homeostasis (Mizushima and Komatsu, 2011). Dysregulation of autophagy is significant for understanding of both the physiological and pathological aspects of several nervous system disorders, including depression. Accumulating evidence from clinical and preclinical studies demonstrated the significant role of autophagy modulation in depression (Jia and Le, 2015; Gassen and Rein, 2019). Therefore, it is important to design a novel treatment strategy for patients with depression by regulating autophagy.

The review focuses on the initiation steps of autophagy, its connection to depression, and its pathological mechanisms. It also summarizes the pharmacological mechanisms of antidepressants by regulating autophagy, providing a scientific basis for their future use in clinical applications.

## 2 The relationship between autophagy and depression

### 2.1 Classification of autophagy

Autophagy is a cellular breakdown process that targets aged organelles or macromolecules, including viruses and bacteria, within eukaryotic cells. It plays a crucial role in alleviating cellular developmental disorders (Levine et al., 2011; Zhou et al., 2020). Autophagy includes three main forms: macro-autophagy, micro-autophagy, and chaperone-mediated autophagy. These forms facilitate the breakdown and recycling of cytosolic components, functioning similarly to lysosomes. Micro-autophagy can be further classified into selective, nonselective, and endosomal forms types, which involve lysosomal depression, lysosomal protrusion, and endosomal depression based on membrane dynamics. The primary mechanism involves the formation of arm- or petal-like protrusions by the lysosomal membrane, which enclose cytoplasmic portions or organelles for degradation (Oku and Sakai, 2018). Chaperone-mediated autophagy, mediated by the chaperone heat shock 70 (HSC70) protein and other proteins, selectively degrades proteins carrying KFERQ-like motifs and transfers them to lysosomes via lysosomal receptors (Dice, 1990). Chaperone-mediated autophagy (CMA) is a crucial process involved in the progression of tumor development, malignant transformation, and neurodegeneration (Gomes et al., 2017).

Macro-autophagy, commonly referred to as autophagy, is the way for cytosolic components to reach lysosomes. It is a distinct multi-step mechanism of membrane transport where cytosolic components and organelles are engulfed and destroyed by double-membrane structures. Autophagy is strictly regulated to maintain an equilibrium between the synthesis and destruction of cellular components, as well as their use and recycling. Abnormal expression of regulatory genes and lysosomal dysfunction can lead to abnormal autophagy. Macro-autophagy is involved in cardiovascular diseases, aging, neurodegenerative diseases, cancer, infectious and inflammatory diseases, and proceeds through initiation, nucleation, extension, fusion, and degradation (Ravanan et al., 2017).

#### 2.1.1 Initiation

Under stress conditions, cells form crescent-shaped bilayer membranes called phagophores in the cytosol, indicating the beginning of autophagy. Bilayer membranes may originate from various sources such as the mitochondrial membrane, endoplasmic reticulum membrane, Golgi membrane, cytoplasmic membrane, ER-mitochondrial contact sites, ER-Golgi intermediates, or recycling endosomes (Ge et al., 2015; Søreng et al., 2018). Autophagy initiation is mediated by unc51-like autophagy-activating kinase 1 (ULK1). Under physiological conditions, mTOR complex 1 (mTORC1) hyperphosphorylates autophagy-related gene (ATG)13 and mammalian ATG1 homologs (ULK1 and ULK2), inhibiting kinase activity of ULK, and preventing the interaction of ATG13 with ULK and FIP200 (a scaffold protein), thus inhibiting autophagy in mammalian cells (Wang et al., 2018a). Stress exposure leads to the dissociation of mTORC1 from the ULK1/ATG1 complex (comprised by ULK1, FIP200, ATG13, and ATG101), allowing ULK1 to anchor to the autophagy precursor structure (PAS), thereby initiating the process of autophagy (Yamamoto et al., 2016).

#### 2.1.2 Nucleation

After the initiation of autophagy, nucleation occurs under the action of vacuum protein sorting 34 (VPS34) and the Beclin-1 complex. VPS34, a type III phosphatidylinositol (PI) kinase, converts PI into PI trisphosphate (PI3P) through phosphorylation. Beclin-1, a pivotal protein within the type III Phosphoinositide 3-kinase (PI3K) complex, regulates autophagosome maturation by binding to VPS3 and its co-factors. Acetylation of VPS34 determines the success or failure of autophagy nucleation. Acetylation at the K29 site hinders the assembly of the core complex including VPS34 and Beclin-1, while acetylation of the K771 site weakens the binding between VPS34 and its substrate, PI. Acetylation of K781 reduces the activity of VPS34 kinase (Su et al., 2017).

#### 2.1.3 Elongation

The expansion of autophagic vacuoles relies on two ubiquitin-like binding systems: the ATG5-ATG12-ATG16L1 conjugation pathway and the ATG8 lipidation pathway. The ATG5-ATG12-ATG16L1 complex plays a crucial role in elongating autophagic vacuoles and acts as a platform for ATG8 lipidation (Ohsumi, 2001; Fujita et al., 2008; Fahmy and Labonté, 2017). This aids in the formation of vesicles surrounded by bilayer membranes. Additionally, the ATG4 enzyme cleaves ATG8 proteins (light chain 3 [LC3], GABA type A receptor–associated protein [GABARAP]l1, and GABARAP, et al.), resulting in the formation of LC3I, which represents the cytosolic variant of LC3. ATG7 then conjugates LC3I to phosphatidylethanolamine, forming LC3II, which remains associated with the autophagosome membrane. The LC3II/LC3I ratio serves as an indicator of autophagic activity (Suzuki et al., 2013).

#### 2.1.4 Fusion and degradation

**FIGURE 1:**
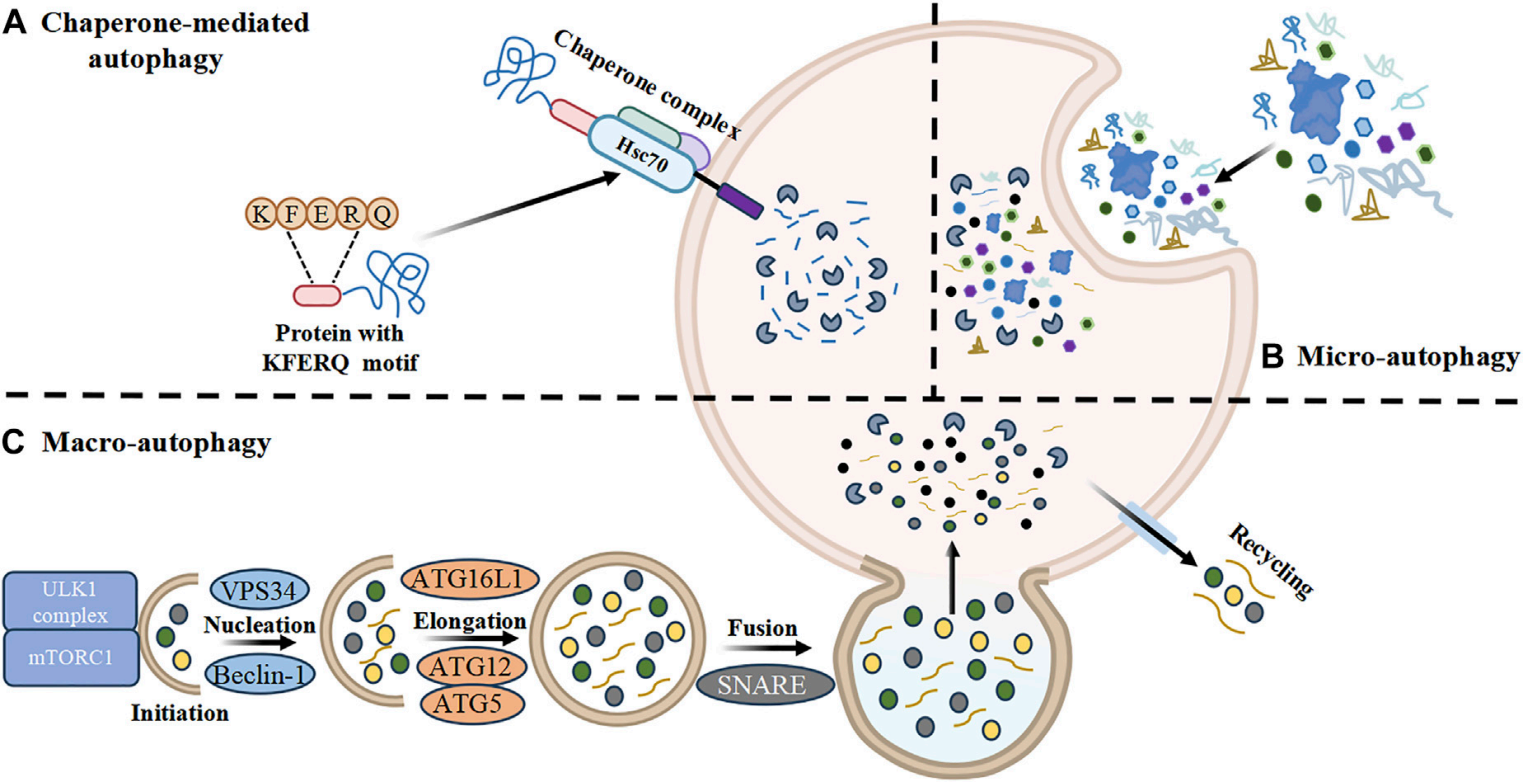
Classification and corresponding processes of autophagy. **(A)** Chaperone-mediated autophagy. Chaperone-mediated autophagy is mediated by the chaperone heat shock 70 (HSC70) protein and other proteins that selectively degrade proteins carrying KFERQ-like motifs and transfer them to lysosomes for autophagy via lysosomal receptors. **(B)** Micro-autophagy. The primary method entails the formation of arm- or petal-like protrusions by the lysosomal membrane, which envelops a portion of the cytoplasm or organelles in order to encase the molecules targeted for degradation. **(C)** Macro-autophagy. Macro-autophagy is the primary method via which cytosolic components reach lysosomes. Macro-autophagy steps include initiation, nucleation, extension, fusion, and degradation. HSC70, Heat shock 70 protein; ULK1, Unc51 like autophagy activating kinase; mTORC1, mTOR complex 1; ATG, Autophagy related genes; SNARE, Soluble NSF attachment protein receptor.

The fusion of autophagosomes with endosomes or lysosomes is a crucial process for the elimination of cellular debris. Studies have demonstrated that the soluble N- ethylmaleimide-sensitive factor (NSF) attachment protein receptor (SNARE) protein family can mediate the fusion of autophagosomal and lysosomal membranes, promoting the maturation of autophagosomes (Shen et al., 2021). Finally, the outer membrane of the autophagosome fuses with the lysosomal membrane to form an autolysosome, allowing lysosomal hydrolases to degrade autophagic cargoes and release the recovered nutrients (such as amino acids and lipids) back into the cytoplasm for reuse (Galluzzi and Green, 2019) (Figure 1).

### 2.2 Autophagy’s role in the development of depression

A growing body of research has established a correlation between autophagy and depression. Dysregulation of autophagy-related gene expression was observed in blood monocytes of patients diagnosed with MDD during a clinical trial (Alcocer-Gómez et al., 2017). Furthermore, abnormal expression of AKT1 and mTOR signaling pathways, which regulate autophagy, has been found in patients with depression (Jernigan et al., 2011; Machado-Vieira et al., 2015). In *postpartum* depression (PPD) patients, changes in extracellular vesicle mRNA, potentially related to autophagy, suggest that interruption of extracellular vesicle mRNA communication may be involved in the pathological development of PPD (Osborne et al., 2022). Bioinformatics techniques have identified potential diagnostic markers for MDD, including autophagy-related genes such as GPR18, PDK4, NRG1, and EPHB2. Additionally, GPR18 may play a role in the pathological progression of MDD (He et al., 2021).

Preclinical investigations have demonstrated the involvement of autophagy in various pathways related to the pathophysiology and progression of depression. There is substantial evidence supporting the association between depression and inflammatory mechanisms (Kohler et al., 2016). Inflammation increases vulnerability to depression, as individuals diagnosed with depression exhibit elevated levels of pro-inflammatory markers, and the utilization of pro-inflammatory medications amplifies the likelihood of depression occurrence (Kohler et al., 2016). Conversely, the administration of antidepressant medication has been observed to decrease peripheral concentrations of inflammatory cytokines (Liu et al., 2020a). The NOD-like receptor pyrin domain-containing 3 (NLRP3) inflammasome is a protein complex that triggers the caspase-1-mediated proteolytic activation of pro-inflammatory cytokines belonging to the interleukin-1β (IL-1β) family, as well as the apoptosis in inflammatory cells, significantly contributing to depression progression (Mangan et al., 2018; Yu et al., 2023). Notably, autophagy is closely linked to the activation of the NLRP3 inflammasome. Dysfunctional lysosomes in the autophagy-lysosome pathway disrupt the degradation of the NLRP3 inflammasome, leading to the generation of pro-inflammatory factors. This process can induce depression-like behavior in mice (Li et al., 2022a). High-mobility group box 1 (HMGB1) has been identified as an early warning protein that induces inflammatory responses after stress exposure (Young et al., 2023). Microglia are a type of cellular component that originates from the mesodermal layer of neural tissue. The primary origin of pro-inflammatory cytokines and inflammation-related proteins under the control of several intracellular signals can be attributed to activated microglia (Park et al., 2023). Activation of the HMGB1/signal transducer and activator of transcription 3 (STAT3)/nuclear factor-kappa B (NF-κB) p65 axis in microglia located in the medial prefrontal cortex (mPFC) facilitates microglial activation and autophagy, contributing to the pathophysiology and progression of depression (Xu et al., 2023). Mechanistic studies have revealed that under physiological conditions, autophagy inhibits excessive activation of the NLRP3 inflammasome and its secreted pro-inflammatory cytokines. Additionally, autophagy plays an important role in the anti-inflammatory process by activating immune cells to produce pro-inflammatory mediators (Zhu and Liu, 2022).

Neurogenesis refers to the process through which neural stem cells (NSCs) or neural progenitor cells (NPCs) generate new neurons. This phenomenon occurs not only during the embryonic and perinatal stages but also in two distinct regions of the mammalian central nervous system: the subventricular zone (SVZ) located in the lateral ventricle and the subgranular zone (SGZ) situated in the dentate gyrus of the hippocampus (Yao et al., 2016). Impaired neurogenesis is believed to underlie the pathogenesis of psychiatric disorders, particularly depression (Kempermann, 2002). In patients with depression, decreased granule cell numbers and volume in the anterior and middle dentate gyrus (DG) were observed, while neurogenesis increased and depressive symptoms improved after antidepressant treatment (Mahar et al., 2014). Autophagy and neurogenesis have also been reported in depression models. Chronic constraint stress is commonly used to induce depression in animal models. Autophagic cell death in NSCs and impaired adult hippocampal neurogenesis have been observed in mice subjected to an induced depression model (Jung et al., 2020). In a corticosterone (CORT)-induced depression model, excessive neuronal autophagy activity was observed in the DG region of the brain, upregulating the expression of ATG5. This led to significant degradation of brain-derived neurotrophic factor (BDNF), which had a detrimental effect on the proliferation of NSCs, NPCs, and neuroblasts. Furthermore, the survival and migration of newly generated immature and mature neurons in the DG were impaired. However, suppression of ATG5 in neurons alleviated these pathological phenomena, leading to an improvement in depression-like behavior in mice (Zhang et al., 2023a). BDNF is a growth factor that plays a crucial role in neuronal development, synapse formation, and synaptic plasticity in the brain (Björkholm and Monteggia, 2016). According to the neurotrophic theory, reduced BDNF expression deprives neurons of necessary nutrition, resulting in neuronal atrophy, decline in synaptic plasticity, and the onset of depression (van Zutphen et al., 2019). Nevertheless, the normalization of BDNF levels plays a significant role in promoting synaptic plasticity, enhancing neuronal repair, and mitigating depression symptoms (Phillips, 2017). Mechanistic studies have shown that a glucocorticoid-induced stress response enhances the expression of the stress-responsive co-chaperone FK506-binding protein 51, activates autophagy, and promotes extracellular BDNF maturation through increased matrix metalloproteinase 9 (MMP9) secretion (Martinelli et al., 2021).

**FIGURE 2:**
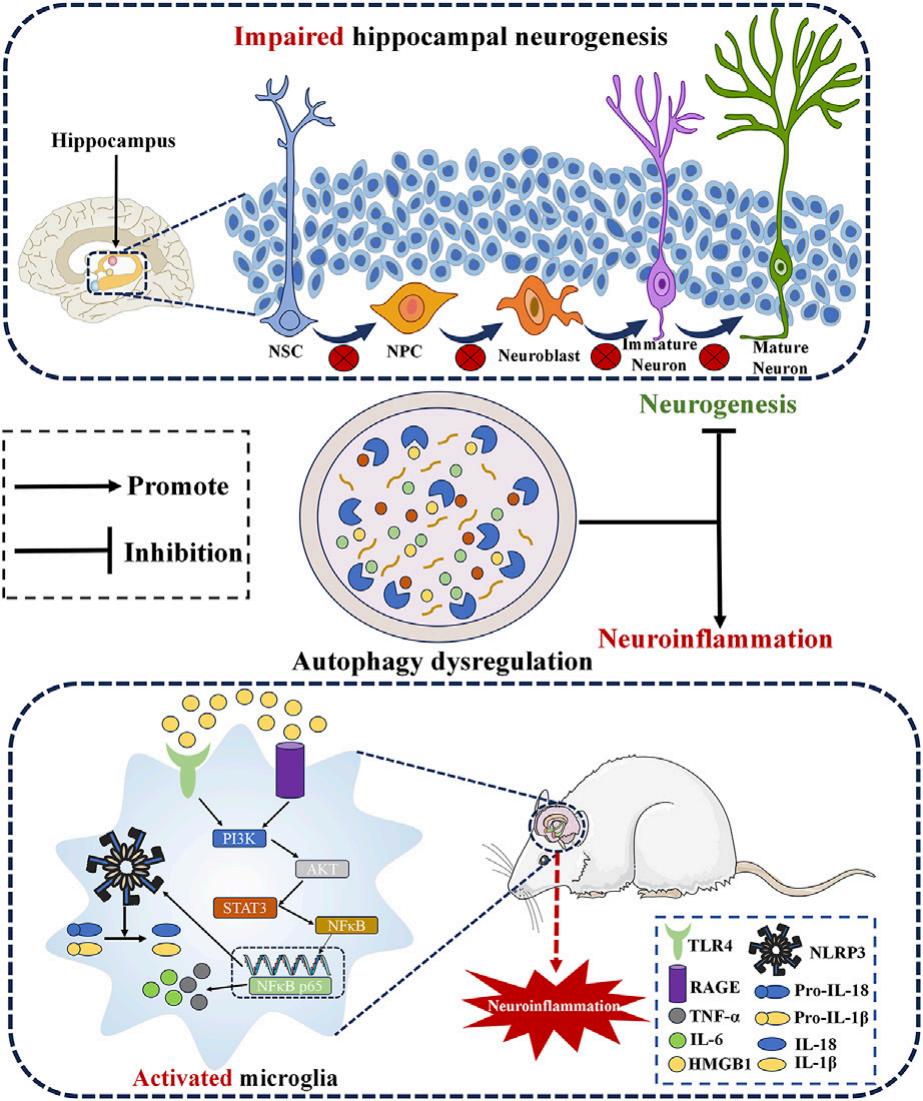
Microscopic pathological mechanism of autophagy induced depression. Autophagy dysregulation participates in the pathological development of MDD by addressing hippocampal neurogenesis and neuroinflammation. NSC, Neural stem cell; NPC, Neural progenitor cell; NLRP3, NOD-like receptor pyrin domain containing 3; IL-1β, Interleukin-1β; HMGB1, High molecular group box 1; NF-κB, Nuclear factor-kappa B; IL-6, Interleukin-6; TNF-α, Tumor necrosis factor-α; TLR4, Toll-like receptors 4; RAGE, advanced glycation end products.

Obesity is characterized by excessive accumulation and storage of body fat, leading to weight gain (Atawia et al., 2019). Numerous reports highlight the association between depression and obesity as a major public health concern (Silva et al., 2020). MDD is particularly common in individuals with severe obesity (i.e., class II-III) (Faulconbridge et al., 2018). In an experimental model of obesity induced by a high-fat diet, it was observed that obesity impairs autophagy by inhibiting the phosphorylation of adenylate-activated protein kinase (AMPK) and promoting the phosphorylation of mTOR. This disruption in cellular signaling pathways contributes to the development of depression-like behavior (Li et al., 2022b) (Figure 2).

## 3 Mechanism of antidepressant chemicals regulating autophagy

### 3.1 Chemicals acting on nervous system diseases

Neurotransmitters in the central nervous system, such as catecholamines (dopamine [DA], norepinephrine [NE], and epinephrine [E]) and indoleamines (serotonin [5-HT]), play a crucial role in emotional regulation, cognition, and sleep (Berke, 2018). Dysregulation of these neurotransmitters can lead to various emotional alterations (Wang et al., 2021a). According to the traditional monoamine hypothesis, depression is caused by a reduction in monoamine neurotransmitters inside the central nervous system. Assessing the levels of these neurotransmitters and their metabolites in the serum can serve as significant diagnostic biomarkers for depression. Pharmacological interventions that increase synaptic concentrations of monoamines have shown efficacy in alleviating depressive symptoms (Wang et al., 2019a). Fluoxetine, a selective SSRI is widely used in clinical practice. Studies have demonstrated that fluoxetine can reverse depressive-like symptoms by activating the nuclear factor erythroid-derived 2-like 2 (Nrf2)-dependent gene expression, reducing neuronal autophagy and cell death in the hippocampus, and mitigating lipopolysaccharide (LPS)-induced peripheral inflammation in mice (Ghosh et al., 2020). Another study found that fluoxetine promotes astrocytic autophagy in a p53-dependent manner and improves mitochondrial damage both *in vivo* and *in vitro* (Shu et al., 2019). Furthermore, fluoxetine therapy has been reported to improve depression-like behavior induced by olfactory bulb resection in rats, reversing hippocampal metabolic disorder and autophagy inhibition (Zhou et al., 2019). PPD is a prevalent psychological condition that occurs after childbirth and poses detrimental effects on maternal wellbeing, with approximately 20% of *postpartum* deaths attributed to suicide resulting from PPD (Payne and Maguire, 2019). Inhibition of autophagy in microglia contributes to the production of inflammation, exacerbating PPD. Fluoxetine has been shown to mediate the autophagy pathway and upregulate the expression of BDNF, offering potential treatment for PPD (Tan et al., 2018).

Agomelatine, a pharmacological compound structurally similar to melatonin, exerts its antidepressant effects by activating melatoninergic receptors (MT1 and MT2) and inhibiting 5-HT2C receptors. These mechanisms contribute to its antidepressant effects (Maddukuri et al., 2021). Research has indicated that agomelatine can regulate neuroinflammation, apoptosis, and autophagy induced by LPS through the inhibition of the G alpha i (2) (Gαi-2)/protein kinase A (PKA)/apoptosis signal-regulating kinase 1 (ASK1) pathway, thereby exhibiting antidepressant properties (Lan et al., 2022). Ketamine, a pharmacological agent acting as a noncompetitive antagonist of the N-methyl-D-aspartate receptor (NMDAR), preferentially inhibits NMDARs containing the GluN2B subunit, mainly found in inhibitory GABAergic interneurons (Sato et al., 2022). Studies have shown that ketamine, even at sub-anesthetic doses (10 mg/kg), exerts an antidepressant effect by inhibiting inflammation and activating autophagy initiation (Lyu et al., 2022). As an emerging mechanism of cellular demise, ferroptosis, is primarily distinguished by cytological alterations. When the ferroptosis pathway is activated, it can trigger depressive symptoms (Mou et al., 2019; Xu et al., 2022). Ketamine has been shown to induce autophagy, improve neuroplasticity, inhibit ferroptosis (Zhang et al., 2022), regulate the autophagic flux of microglia through the HMGB1-advanced glycation end products (RAGE) receptor pathway, and modulate microglial polarization (Wu et al., 2022).

### 3.2 Chemicals regulating endocrine metabolism

Carbagliflozin, a sodium glucose cotransporter 2 (SGLT2) inhibitor used as an antidiabetic drug, has gained attention due to its additional cardiovascular benefits (Du et al., 2022). In a recent preclinical investigation, the efficacy of canagliflozin in ameliorating depression-like behavior induced by chronic unexpected mild stress (CUMS) in rats was examined. The results revealed that canagliflozin modulates the AMPK/mTOR autophagy signaling pathway, exhibits anti-inflammatory and neuroprotective effects, and alleviates depressive symptoms (Khedr et al., 2023).

Rosiglitazone, a thiazolidinedione (TZD) used as an antidiabetic drug because of its insulin sensitivity, acts by activating the intracellular receptor class of peroxisome proliferator-activated receptor gamma (PPARγ) (Fryklund et al., 2022). Previous studies have shown the effectiveness of rosiglitazone in alleviating depression-like symptoms in animal models (Sharma et al., 2012; Zong et al., 2018). Additionally, rosiglitazone improves dexamethasone-induced depression in mice through the regulation of cerebral glucose metabolism and the AMPK/mTOR signaling pathway (Alhaddad et al., 2023). The mTOR signaling pathway plays a crucial role in the control of autophagy. A comprehensive investigation utilizing both *in vivo* and *in vitro* approaches elucidated the underlying mechanism by which rosiglitazone exerts therapeutic effects on depression. It was found that rosiglitazone promotes neuroprotection by upregulating autophagy and inducing excessive apoptosis in astrocytes affected by depression (Zhao et al., 2017).

Metformin, the first-line therapy for type 2 diabetes, mediates blood glucose control through hepatic gluconeogenesis and has pleiotropic effects on glucose metabolism (LaMoia and Shulman, 2021). Metformin also plays a significant role in the therapeutic management of depression. Studies have reported that metformin can modulate microbiota-derived inosine levels, ameliorate anxiety and depression-like withdrawal symptoms caused by methamphetamine in mice (Yang et al., 2022a), and potentially reduce the likelihood of depressive symptoms compared to other oral hypoglycemic medications (Yu et al., 2022a). In a recent study, metformin was found to improve depression-like behavior in a mouse model of Parkinson’s disease by increasing protein expression in the autophagy signaling pathway and promoting autophagosome formation (Mendonça et al., 2022).

Atorvastatin, a statin lipid regulator, inhibits cholesterol production, resulting in reduced blood cholesterol levels and decreased cardiovascular risk (Yu et al., 2022b). Previous studies have demonstrated that atorvastatin can prevent LPS-induced depression-like behavior (Taniguti et al., 2019) and activate autophagy while relieving oxidative stress through acting on NADPH oxidase 2 (NOX2), thereby improving depression-like behavior in mice with Parkinson’s disease (Yan et al., 2020).

### 3.3 Other types of chemicals

Hydrogen sulfide (H_2_S) is an endogenous gaseous transmitter that can be produced internally in mammals through four enzymatic pathways (Wu et al., 2018). Intervention of H_2_S is used to treat depression as it counteracts the depressive and anxiety-related effects caused by sleep deprivation (Kang et al.). It achieves this by inhibiting neuroinflammation via a silent mating-type information regulation 2 homolog 1 (SIRT1)-dependent mechanism (Kang et al.). Furthermore, studies have indicated that inhibiting inflammation and ferroptosis may potentially alleviate depression-like behavior in rats with type 1 diabetes (Wang et al., 2021b). H_2_S has been reported to alleviate depressive behavior by increasing adiponectin levels, thereby improving hippocampal synapse formation dysfunction and excessive autophagy (Tian et al., 2018). Moreover, the antidepressant properties of H_2_S are attributed to its ability to enhance the activity of the brain-derived neurotrophic factor-tropomyosin-related kinase B (TrkB) pathway in the hippocampus, thus facilitating autophagy (Liu et al., 2020b).

Roflumilast, a highly effective and specific inhibitor of phosphodiesterase-4 (PDE4), has shown potential in reducing exacerbations in individuals suffering from severe chronic obstructive pulmonary disease (COPD) accompanied by chronic bronchitis or a history of exacerbation (Wedzicha et al., 2016). Roflumilast activates the AMPK/mTOR/ULK1 autophagy pathway and provides neuroprotective effects in the treatment of depression (Zaki et al., 2023). Resolvin D1 (RvD1) is a lipid mediator with notable anti-inflammatory properties derived from docosahexaenoic acid (DHA), an omega-3 polyunsaturated fatty acid, synthesized endogenously in the organism (Roohbakhsh et al., 2022). Studies in mice have demonstrated that RvD1 induces microglial autophagy, suppresses M1 polarization and inflammatory response, reduces neurotoxicity, and ameliorates depression-like behavior (Xiong et al., 2023).

Bafilomycin A1, an organic macrolide antibiotic derived from *Streptomyces* griseus, specifically inhibits vacuolar H^+^-ATPase, impeding the acidification process in organelles housing this enzyme (Xu et al., 2020). The antidepressant properties of Bafilomycin A1 are attributed to its ability to counteract apoptosis, autophagy, and neuroinflammation in the hippocampus (Wang et al., 2018b). Melatonin, a hormone of the indole class synthesized in the pineal gland via the tryptophan-serotonin biosynthetic pathway, is regulated by the brain’s circadian clock (Vasey et al., 2021). Melatonin exhibits antidepressant effects in an LPS-induced animal depression model, and its mechanism involves regulating autophagy through the Forkhead box o (FOXO) 3a signaling pathway (Ali et al., 2020).

Vitamin E (VE), an essential vitamin discovered in the 1920s, is widely used for its antioxidative properties. VE encompasses a group of eight lipid-soluble molecules, including alpha, beta, gamma, and delta forms of tocopherols, with alpha-tocopherol being the predominant variant (Miyazawa et al., 2019; Yang et al., 2020). Alpha -Tocopherol has been proven to promote autophagy in mice subjected to CUMS through the AMPK/mTOR pathway, thereby mediating antidepressant effects (Huang et al., 2018a) (Table 1).

**TABLE 1:** Regulation of antidepressant chemicals on autophagy.

Antidepressant chemical	Chemical structure	Regulating autophagy mechanism	References
Fluoxetine	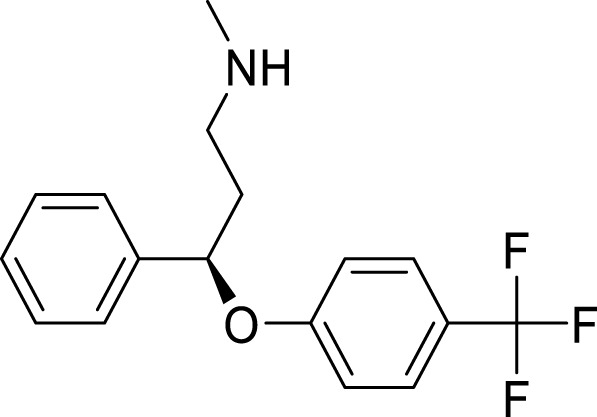	Activating the hippocampal Nrf2 pathway to reduce autophagy activity and alleviate cell death	Shu et al. (2019)
		Promote autophagy, alleviate mitochondrial damage, and alleviate the pathological damage of hippocampal astrocytes	Shu et al. (2019)
		Activating hippocampal autophagy and improving hippocampal metabolic disorders	Zhou et al. (2019)
		Mediates antidepressant via autophagy pathway and upregulates BDNF levels	Tan et al. (2018)
Agomelatine	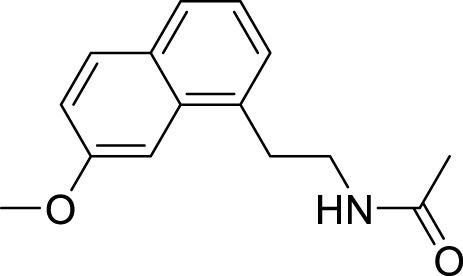	Inhibition of Gαi-2/PKA/ASK1 pathway activity to anti inflammation and regulate autophagy activity	Lan et al. (2022)
Ketamine	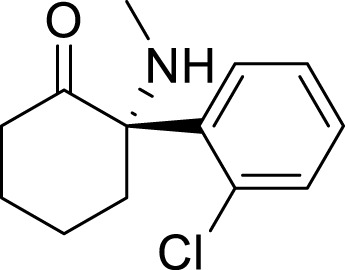	Inhibit inflammation and activate autophagy	Lyu et al. (2022)
		Triggering autophagy, improving neuroplasticity, and inhibiting ferroptosis	Zhang et al. (2022)
		Regulates the autophagic flux of microglia and microglial polarization through the HMGB1/RAGE pathway	Wu et al. (2022)
Carbagliflozin	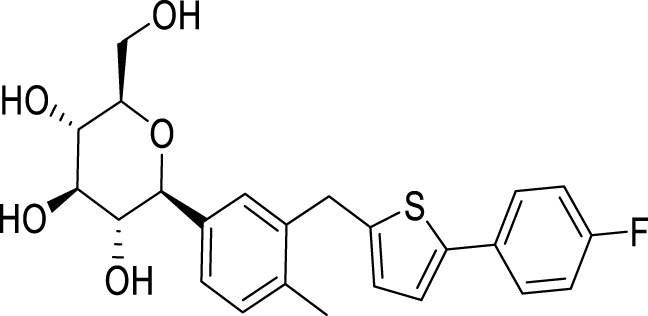	Regulating AMPK/mTOR autophagy signaling pathway and its anti-inflammatory and neuroprotective effects	Khedr et al. (2023)
Rosiglitazone	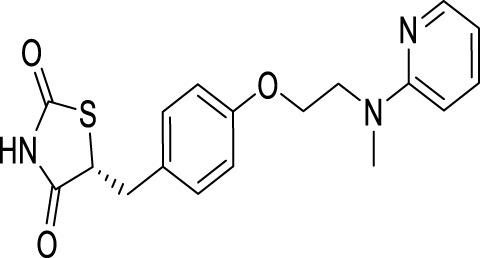	Regulation of brain glucose metabolism and AMPK/mTOR signaling pathway	Alhaddad et al. (2023)
		Upregulate autophagy level to exert neuroprotective effect and alleviate excessive apoptosis of astrocytes	Zhao et al. (2017)
Metformin	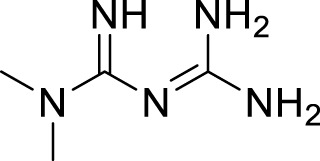	Increase protein expression of autophagy signaling pathways and promote autophagosome formation	Mendonça et al. (2022)
Atorvastatin	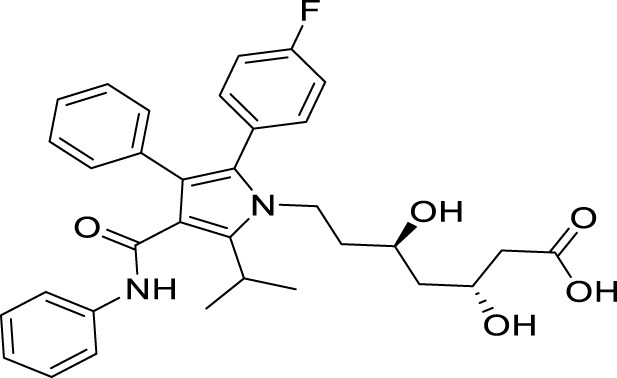	Acting on NOX2 to activate autophagy and alleviate oxidative stress	Yan et al. (2020)
H_2_S	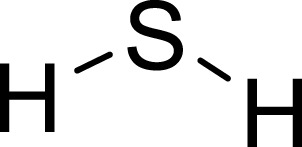	Upregulation of adiponectin levels, improvement of hippocampal synaptic dysfunction, and relief of excessive autophagy	Tian et al. (2018)
		Enhancing the activity of hippocampal BDNF/TrkB signaling pathway to promote autophagy	Liu et al. (2020b)
Roflumilast	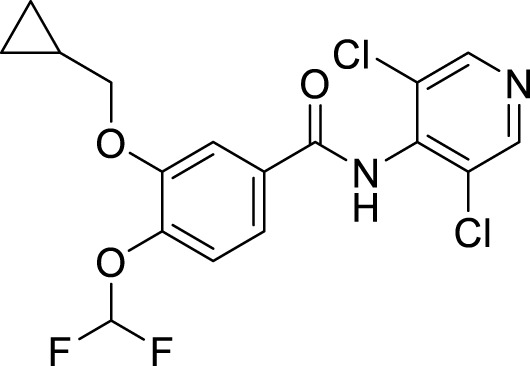	Activating the AMPK/mTOR/ULK1 autophagy pathway and exerting neuroprotective effects	Zaki et al. (2023)
Resolvin D1	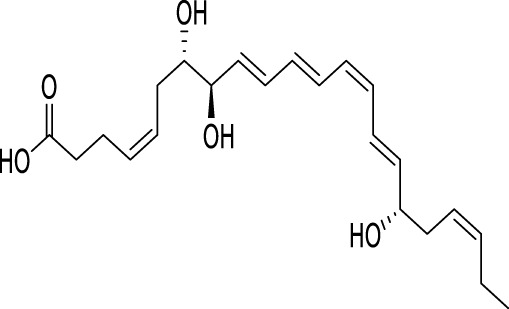	Promote autophagy of microglia, inhibit M1 polarization and inflammatory response, and reduce neurotoxicity	Xiong et al. (2023)
Bafilomycin A1	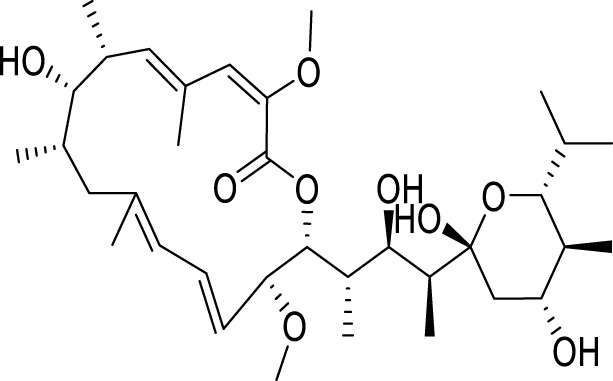	Regulating cell apoptosis, autophagy, and neuroinflammation in the hippocampus	Wang et al. (2018b)
Melatonin	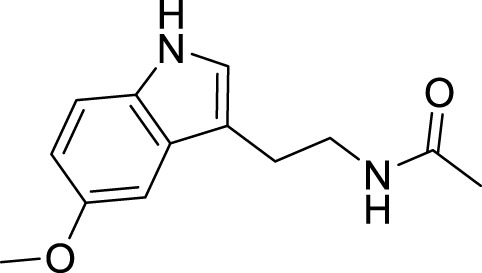	Autophagy activity regulating the FOXO3a signaling pathway	Ali et al. (2020)
α- tocopherol	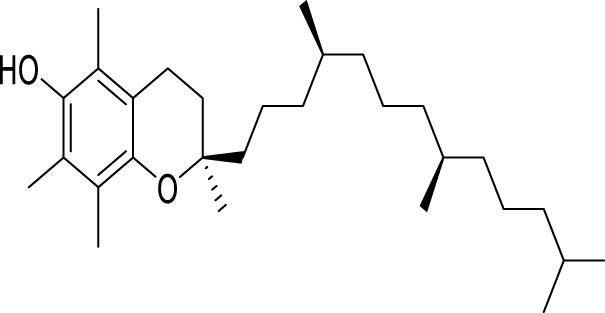	Promoting autophagy by acting on the AMPK/mTOR pathway	Huang et al. (2018a)

Nrf2, nuclear factor (erythroid-derived 2)-like 2; BDNF, brain-derived neurotrophic factor; Gαi-2/PKA/ASK1, G alphai (2)/protein kinase A/apoptosis signal-regulating kinase 1; HMGB1/RAGE, High molecular group box 1/advanced glycation end products; AMPK/mTOR, adenylate-activated protein kinase/mammalian target of rapamycin; TrkB, brain-derived neurotrophic factor-tropomyosin-related kinase B; ULK1:unc51 like autophagy activating kinase 1; FOXO3a, Forkhead box O 3a.

## 4 Treatment of depression by regulating autophagy using TCM

### 4.1 Active compounds of TCM

Resveratrol, a phenolic compound originally derived from Veratrum grandiflorum, is abundantly present in grapes, wine, peanuts, soybeans, and berries. Its therapeutic potential in the context of depression has attracted significant attention from researchers and medical professionals (Breuss et al., 2019). Extensive research has been conducted on the use of resveratrol for treating depression (Moore et al., 2018). Studies have found that CUMS can inhibit the activity of the SIRT1 signaling pathway in mice, resulting in downregulation of autophagy and mitophagy-related protein expression, and neuronal damage. However, treatment with resveratrol can alleviate these pathological phenomena (Tabassum et al., 2023). In a mouse model of PPD, intragastric administration of resveratrol alleviated depressive behavior by stimulating SIRT1, inducing autophagy, and inhibiting the AKT/mTOR signaling pathway (Ye et al., 2023).

Oridonin is the principal bioactive constituent within the Chinese botanical remedy *Rabdosia rubescens*, demonstrating significant anti-inflammatory properties. It exhibits considerable anticancer activities by inducing cell cycle arrest andapoptosis, and inhibiting angiogenesis (He et al., 2018). Previous studies have shown that oridonin can regulate the signal of PPAR-γ and α-amino-3-hydroxy-5-methyl-4-isoxazole propionic acid glutamate receptors (AMPARs) in the prefrontal cortex to treat depression (Liu and Du, 2020). Recent studies have found that the antidepressant effect of oridonin involves blocking the interaction between NLRP3 and NIMA-related kinase 7 (NEK7) to inhibit neuroinflammation and autophagy injury (Liang et al., 2022). Additionally, oridonin can inhibit the NLRP3 inflammasome by activating autophagy to alleviate depressive symptoms caused by LPS (Li et al., 2022c). Previous studies have also demonstrated that *Scutellaria baicalensis* exerts an antidepressant effect by reducing the expression of LC3-B (a marker of the autophagy pathway) in neurons of the hippocampal CA1 region (Li et al., 2021). Baicalin, a flavonoid derived from the desiccated roots of *S. baicalensis*, exhibits diverse pharmacological properties (Shen et al., 2019). It intervenes in depression through multiple targets and channels (Liu et al., 2019). Importantly, baicalin enhances Nip-like protein (NIX)-mediated mitophagy by activating the AMPK/peroxisome proliferator-activated receptor-gamma coactivator (PGC)-1α pathway to treat depression (Jin et al., 2023).

*Morinda officinalis*, a type of TCM grown in Southeast China, effectively strengthens bones, tonifies the kidneys, and treats impotence, menstrual disorders, and inflammatory diseases. *Morinda officinalis oligosaccharide* is one of its main effective components that can alleviate depression-like behavior by regulating intestinal microbes (Chi et al., 2020). Interestingly, in an animal model of hypertension with depression, *M. officinalis oligosaccharide* increased the expression of mitofusion 2 (Mfn2) to activate mitophagy mediated by the PI3K/AKT/mTOR pathway, thereby playing a protective role on astrocytes (Yang et al., 2023). *Andrographis paniculata* is a traditional herbal medicine commonly used in Asian countries to relieve symptoms caused by colds (Burgos et al., 2020). Andrographolide is one of its active ingredients and has anti-inflammatory, antitumor, antiviral, and antifibrotic effects (Zhang et al., 2021a). More importantly, andrographolide activates autophagy to inhibit inflammation and improve depression-like behavior induced by CUMS in mice (Geng et al., 2019).

Allicin, a naturally occurring compound found in the bulbs of plants belonging to the Liliaceae family, has been studied for its potential therapeutic properties, including anticancer, antihypertensive, hypoglycemic, and lipid-lowering effects (Shi et al., 2019). It can also alleviate depression-like symptoms caused by a high-fat diet. Its mechanism involves improving mitochondrial function to regulate autophagy, relieve oxidative stress, and optimize NOX/Nrf2 imbalance, thereby reducing insulin resistance in the hippocampus (Gao et al., 2019). Salvianolic acid B, a phenolic acid derived from the desiccated roots and rhizomes of *Salvia miltiorrhiza*, has extensive usage in managing cardiovascular and cerebrovascular ailments (Li et al., 2020). It also plays an important role in the nervous system, particularly in depression. Studies have demonstrated that salvianolic acid B enhances autophagy and facilitates the elimination of the NLRP3 inflammasome, thereby eliciting neuroprotective and antidepressant effects (Jiang et al., 2017).

*Patchouli alcohol*, a tricyclic sesquiterpene, is a natural compound found in *Pogostemon cablin* that possesses various beneficial pharmacological effects (Lee et al., 2020). The activation of the mTOR signaling pathway plays a crucial role in regulating autophagy and exerting antidepressant effects (Zhuo et al., 2020). *Lotus plumule*, which refers to the green embryo found in lotus seeds, is a traditional medicinal substance commonly consumed in China as tea. It is believed to possess properties that can alleviate symptoms of irritability and hypertension (Xiong et al., 2016). Network pharmacology, distinguished by its emphasis on integrity and systematicity, utilizes high-throughput screening, network visualization, and analysis to explore intricate connections between drugs, targets, and diseases. This approach proves advantageous in advancing the research and development of TCM (Wang et al., 2021c). Using network pharmacology and experimental verification, Chen et al. discovered that bioactive alkaloids from *Lotus plumule* inhibit neuroinflammation and alleviate LPS-induced depressive behavior by mediating BDNF-driven endoplasmic reticulum (ER) stress and autophagy (Chen et al., 2019). Quercetin, a flavonoid compound possessing antioxidant, antiviral, antibacterial, and anti-inflammatory properties, is abundantly found in various fruits and vegetables (Di Petrillo et al., 2022). Studies have shown that the antidepressant effect of quercetin is the result of protecting neurons by promoting mitophagy to inhibit the activation of the NLRP3 inflammasome mediated by mitochondrial reactive oxygen species (mtROS) in microglia (Han et al., 2021).

*Euryale ferox*, a plant with a long history of use in TCM, has primarily been employed to enhance renal function, invigorate vital essence, and strengthen the spleen to alleviate symptoms of diarrhea. This treatment modality is frequently observed in managing various medical conditions, including spermatorrhea, gonorrhea, dysmenorrhea, urinary incontinence, and fecal incontinence (Jiang et al., 2023). The petroleum ether fraction of *E. ferox* activates autophagy through the regulation of the AMPK pathway, exhibiting therapeutic effects in animal models of depression (Huang et al., 2018b). Apigenin, a flavonoid widely present in fruits and vegetables, is associated with numerous health advantages (Majma Sanaye et al., 2022). Similarly, apigenin has been shown to promote autophagy and improve depression through the AMPK/mTOR signaling pathway (Zhang et al., 2019).

Ginsenoside Rg1, a protopanaxatriol saponin, is abundantly found in ginseng products and extensively investigated in the context of endocrine disorders (Liu et al., 2017). The antidepressant mechanism of Ginsenoside Rg1 involves the effects on ubiquitin-proteasome and autophagy-lysosome degradation pathways of connexin 43 (Cx43) (Wa et al., 2021). Aconite and its active components are commonly used for treating depression (Liu et al., 2012). The coalescence of aggregate alkaloids found in aconite and ginsenosides regulates autophagy and hippocampal synaptic plasticity through the activation of the BDNF-mTORC1 signaling pathway, contributing to the manifestation of an antidepressant effect (Jin et al., 2022). Silibinin, an active compound extracted from Compositae *plant milk thistle*, has various pharmacological effects, including anti-inflammatory, antioxidant, and antifibrotic activities (Jiang et al., 2021). Studies have provided evidence indicating that silibinin mitigates neuronal damage by modulating the BDNF/TrkB pathway while reducing the extent of autophagy in the hippocampus (Song et al., 2017). *Radix Polygalae*, a renowned Chinese herbal medicine, has been utilized in China for numerous purposes over several centuries, including as an expectorant, tonic, sedative, and antipsychotic agent (Jiang et al., 2021). The extract inhibits neuroinflammation and treats depression by promoting autophagy (Zhou et al., 2021) (Table 2).

**TABLE 2:** Regulation of autophagy by the active compounds of traditional Chinese medicine.

Active compounds of TCM	Chemical structure	Animal type	Dosage and usage	Duration of the study	Behavioral testing evaluation	Antidepressant mechanisms	References
Resveratrol	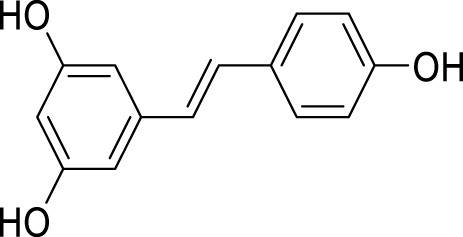	C57BL/6 mice	30 mg/kg Injected intraperitoneally	21 days	OFT, EPM, FST, SPT, TST	Regulating the activity of SITR1 signaling pathway and regulating the expression of autophagy proteins	Tabassum et al. (2023)
		C57BL/6 mice	20 mg/kg Injected intraperitoneally	28 days	OFT, TST, FST	Stimulating SIRT1, inducing autophagy and inhibiting AKT/mTOR signaling pathway activity	Ye et al. (2023)
Oridonin	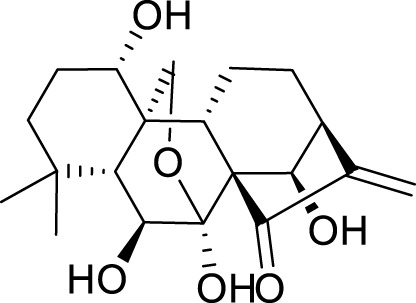	Sprague–Dawley rats	5, 10, 20 mg/kg Gavage	6 weeks	SPT, FST	Inhibiting the interaction between NLRP3 and NEK7 to alleviate neuroinflammation and autophagy damage	Liang et al. (2022)
		C57BL/6 mice	20 mg/kg Gavage	14 days	SPT, FST, TST	Activating autophagy to inhibit NLRP3 inflammasome activity	Li et al. (2022c)
Baicalin	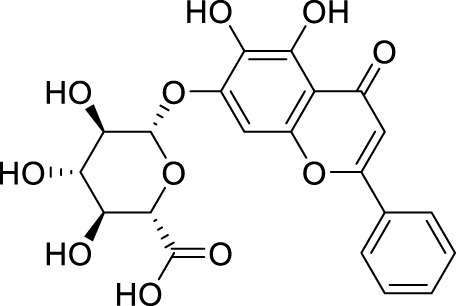	C57BL/6 mice	20 mg/kg Gavage	6 weeks	SPT, TST	Activating the AMPK/PGC-1αpathway to enhance NIX mediated mitochondrial autophagy	Jin et al. (2023)
Andrographolide	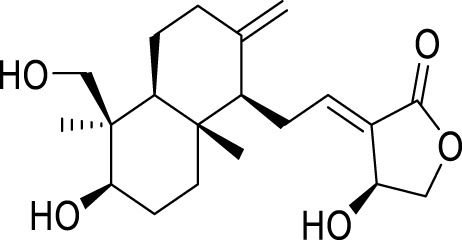	C57BL/6 mice	2, 5, 5 mg/kg Gavage	46 days	FST, TST, SPT, Y-maze	Activating autophagy to suppress inflammation	Geng et al. (2019)
Allicin	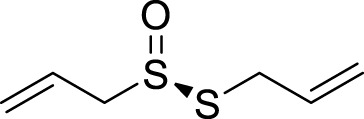	C57 mice	50, 100, 200 mg/kg Gavage	15 weeks	SPT, OFT, TST	Improving mitochondrial function to regulate autophagy, alleviate oxidative stress, and improve NOX/Nrf2 disorder	Gao et al. (2019)
Salvianolic acid B	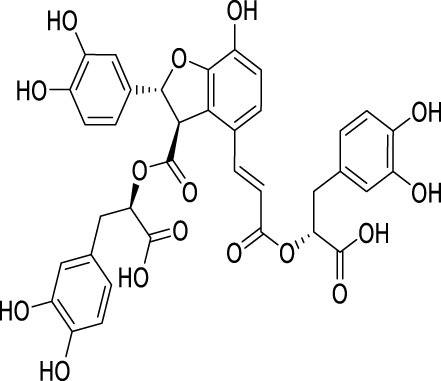	Sprague–Dawley rats	20 mg/kg Injected intraperitoneally	14 days	FST, SPT, EPM	Promoting autophagy and inducing clearance of NLRP3 inflammasomes	Jiang et al. (2017)
Patchouli alcohol	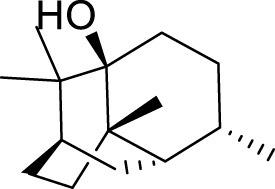	Sprague–Dawley rats	10, 20, 40 mg/kg Gavage	8 weeks	OFT, SPT, FST	Activating the mTOR signaling pathway to regulate autophagy	Zhuo et al. (2020)
Quercetin	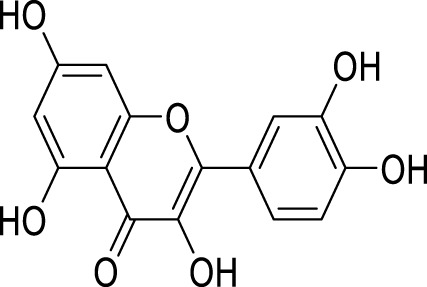	C57BL/6 mice	30, 60 mg/kg Injected intraperitoneally	9 days	TST, FST	Promoting mitochondrial autophagy to inhibit mtROS mediated NLRP3 inflammasome activation in microglia	Han et al. (2021)
Apigenin	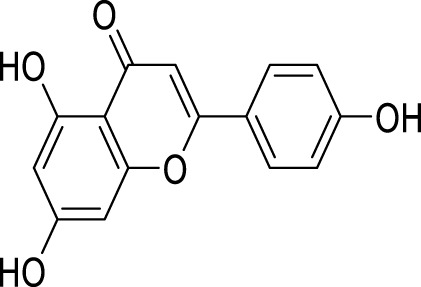	BALB/c mice	20, 40, 60 mg/kg Injected intraperitoneally	21 days	SPT, OFT, FST, TST	Acting on the AMPK/mTOR signaling pathway to promote autophagy	Zhang et al. (2019)
Ginsenoside Rg1	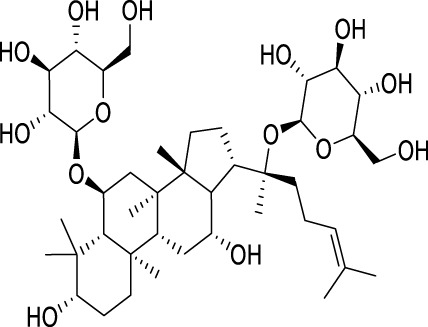	Primary astrocytes (Isolation from Sprague Dawley rats)	0.1, 1, 10 μM	Not Applicable	Not Applicable	Regulating the ubiquitin proteasome and autophagy lysosomal degradation pathways of Cx43	Wa et al. (2021)
Silibinin	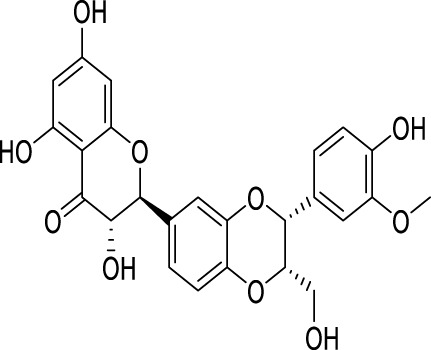	Sprague–Dawley rats	25, 50, 100 mg/kg Gavage	15 days	TST, EPM, FST	Relieve neuronal damage and reduce autophagy in the hippocampus through the BDNF/TrkB pathway	Song et al. (2017)

SIRT1, silent mating-type information regulation 2 homolog 1; AKT/mTOR, adenylate-activated protein kinase/mammalian target of rapamycin; NLRP3, NOD-like receptor pyrin domain containing 3; NEK7, NIMA-related kinase 7; AMPK, adenylate-activated protein kinase; PI3K, Phosphoinositide 3-kinase; Mfn2, mitofusion 2; PGC-1α:peroxisome proliferator-activated receptor-gamma coactivator-1α; NIX, Nip-like protein; NOX, NADPH, oxidase; mtROS, mitochondrial reactive oxygen species; Cx43, connexin 43; OFT, open field test; TST, tail suspension test; FST, forced swimming test; SPT, sucrose preference test; EPM, elevated plus maze.

### 4.2 TCM compounds

Xiaoyaosan (XYS) is a TCM formulation documented in the monograph titled “Prescription of the Taiping People’s Welfare Pharmacy Bureau” during the Northern Song Dynasty (960–1127 AD). It consists of Chaihu (*Radix Bupleuri*), Danggui (*Radix Angelicae Sinensis*), Baishao (*Radix Paeoniae Alba*), Baizhu (*Rhizoma Atractylodis Macrocephalae*), Fuling (*Poria*), Bohe (*Herba Menthae Haplocalyx*), Shengjiang (*Rhizoma Zingiberis*), and Gancao (*Radix Glycyrrhizae*). Currently, various strategies are available for the treatment of depression. TCM compounds, including XYS, have demonstrated antidepressant effects in both clinical and preclinical studies (Wang et al., 2023). In a recent report, XYS was found to regulate autophagy and the expression of glucose transporter-4 (GLUT4) in hypothalamic neurons of depressed mice (Yang et al., 2022b). Moreover, modified XYS alleviated neuronal apoptosis by triggering autophagy and effectively treated depression-like behavior caused by CUMS (Wang et al., 2019b). Another study discovered that modified XYS inhibits M1 polarization of microglia and alleviates neuroinflammation by activating the PI3K/AKT/mTOR pathway to induce autophagy (Su et al., 2023). The MingmuXiaoyao granule, a modified compound derived from XYS, has been observed to regulate autophagy through modulation of the PI3K/AKT/mTOR signaling pathway, thereby enhancing retinal morphology and function, as well as alleviating depression-like behavior in rats subjected to CUMS (Ma et al., 2022).

Lily Bulb and Rehmannia Decoction is a specialized medicinal formulation utilized for the therapeutic management of “lily disease,” characterized by symptomatology similar to clinical depression (Zhang et al., 2020). Metabolomics analyzes metabolites in biological cells or tissues, identifies abnormal metabolic networks associated with diseases, analyzes data collected by instruments through multivariate statistical methods to identify differential metabolites and describe changes in metabolic pathways. This approach helps explain the response mechanism of organisms to corresponding stimuli (Johnson et al., 2016). The integration of network pharmacology and metabolomics offers a promising approach for comprehensively unraveling the therapeutic mechanisms underlying TCM in the context of affective disorders such as depression (Liu et al., 2021; Qu et al., 2021). Chi et al. demonstrated that treatment with Lily Bulb and Rehmannia Decoction alleviated LPS-induced depression-like behavior in rats. They also found that autophagy signaling pathway regulation contributes to its antidepressant effect through the integration of network pharmacology and metabolomics (Chi et al., 2022).

Kaixinsan is a TCM compound first proposed by Sun Simiao in the *Tang* Dynasty in the “Golden Prescriptions of the Northern Ages.” It is composed of *Polygala tenuifolia*, *Ginseng*, *Poria cocos*, and *Acorus tatarinowii*. Kaixinsan has long been used as a classic formula for treating depression (Wang et al., 2022a; Jiao et al., 2022). Its antidepressant effects have been demonstrated both *in vivo* and *in vitro* by activating autophagy and suppressing NLRP3-mediated inflammation (Yu et al., 2021).

**FIGURE 3:**
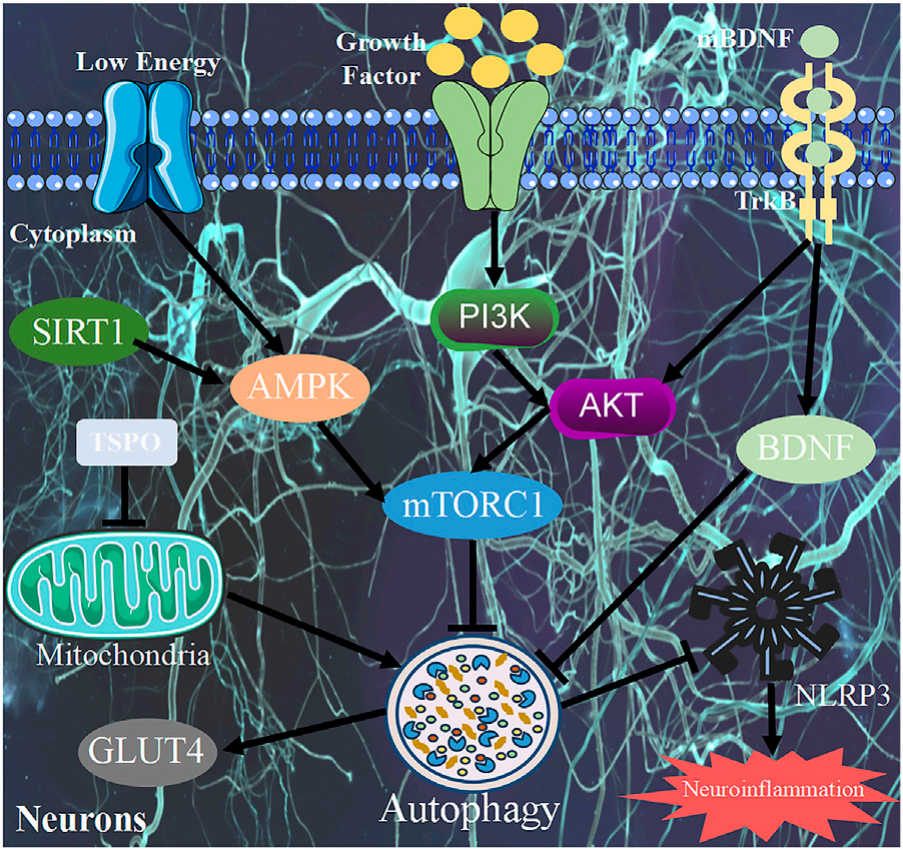
Pharmacological mechanism of TCM in regulating autophagy. TCM, Traditional Chinese medicine; mTORC1, mTOR complex 1; BDNF, Brain-derived neurotrophic factor; TrkB, Brain-derived neurotrophic factor-tropomyosin-related kinase B; TSPO, 18 kDa translocator protein; PI3K, Type III Phosphoinositide 3-kinase; AMPK, Adenylate-activated protein kinase; GLUT4, Glucose transporter-4; AKT, Adenylate-activated protein kinase; SIRT1, Silent mating-type information regulation 2 homolog 1.

Sinisan, derived from *Zhang Zhongjing*’s treatise on febrile diseases, has been a famous TCM formula for treating depression for thousands of years (Zhang et al., 2023b). It consists of Chaihu (*Radix Bupleuri*), Shaoyao (*Paeonia lactiflora*), Zhiqiao (*Fructus aurantii Immaturus*), and Gancao (*Radix Glycyrrhizae*). Sinisan has been widely used in China to treat liver depression, spleen deficiency, digestive system diseases, and depression (Wang et al., 2022b). Sinisan was shown to prevent excessive autophagy by activating the PI3K/AKT/mTOR pathway, providing a neuroprotective role in a model of CORT-induced neurotoxicity. Thus, it exhibits potential therapeutic effects on depression (Zhang et al., 2021b). The prescription known as Wulingsan, initially documented in the Treatise on Febrile Diseases, has traditionally been employed as a therapeutic intervention for addressing water retention resulting from bladder gasification. This prescription has gained significant popularity in the treatment of ascites (Mou et al., 2022). Studies have found that Wulingsan has obvious antidepressant effects, and its potential mechanism of action involves improving the mitophagy signaling pathway mediated by the 18 kDa translocator protein (TSPO) (Li et al., 2016) (Table 3; Figure 3).

**TABLE 3:** Regulation of autophagy by the antidepressant compounds of traditional Chinese medicine.

TCM compounds	Modeling method	Animal type	Dosage	Duration of the study	Behavioral testing evaluation	Antidepressant mechanisms	References
Xiaoyaosan	CUMS	C57BL/6 mice	0.658 g/kg/d	13 weeks	OFT, SPT, TST	Regulating autophagy and GLUT4 expression in hypothalamic neurons	Yang et al. (2022b)
Modified Xiaoyaosan	CUMS	C57 mice	23 g/kg/d	6 weeks	SPT, TST, OFT, FST	Activate neuronal autophagy to alleviate neuronal damage	Wang et al. (2019b)
Modified Xiaoyaosan	LPS	ICR mice	3.8, 7.6 g/kg/d	16 days	SPT, TST, OFT	Activating the PI3K/Akt/mTOR pathway triggers autophagy to inhibit M1 polarization of microglia and alleviate neuroinflammation	Su et al. (2023)
Mingmu Xiaoyao granule	CUMS	Sprague–Dawley rats	3.8, 7.6 g/kg/d	12 weeks	SPT, OFT	Regulating autophagy through the PI3K/Akt/mTOR signaling pathway	Ma et al. (2022)
Lily bulb and Rehmannia decoction	LPS	Sprague–Dawley rats	90 g/kg	17 days	SPT, FST, EPM	The mechanism of action involves regulation of autophagy pathways	Chi et al. (2022)
KaiXinSan formula	CUMS	Wistar rats	3, 5, 10 g/kg/d	47 days	SPT, OFT, FST	Regulating autophagy to suppress NLRP3 mediated inflammation	Yu et al. (2021)
Sinisan	CORT	Sprague–Dawley rats	0.49 g/mL	Not Applicable	Not Applicable	Activating the PI3K/AKT/mTOR pathway to prevent excessive autophagy	Zhang et al. (2021b)
Wuling powder	IS	ICR mice	0.5, 1, 2 g/kg	2 weeks	NSFT, FST	Regulation of TSPO mediated mitochondrial autophagy signaling pathway	Li et al. (2016)

CUMS, chronic unpredictable mild stress; LPS, lipopolysaccharide; CORT, corticosterone; IS, inescapable e-shock; GLUT4, glucose transporter-4; PI3K/Akt/mTOR, Phosphoinositide 3-kinase/adenylate-activated protein kinase/mammalian target of rapamycin; TSPO:18 kDa translocator protein; OFT, open field test; TST, tail suspension test; FST, forced swimming test; SPT, sucrose preference test; NSFT, Novelty-suppressed feeding test.

## 5 Strengths and limitations

MDD is an ongoing challenge in modern medicine since its pathogenesis has not been fully understood and there is still a lack of strategies that can successfully prevent or completely reverse its occurrence (Chen et al., 2022). Autophagy plays a crucial role in MDD, making the regulation of autophagy a potential strategy for the prevention of depression. For the first time, this review provided a comprehensive summary of the mechanisms by which different antidepressant medications, such as fluoxetine, agomelatine, and ketamine, as well as other chemicals, regulate autophagy to treat MDD. However, some antidepressant chemicals that act on the nervous system, such as SSRIs, have been found to have adverse effects such as nausea, headache, chronic sexual dysfunction, and weight gain. Most treatments have delayed effects and high rates of no response (Wang et al., 2019a; Qu et al., 2021; Wei et al., 2022). Ketamine can cause hallucinations, hepatotoxicity, neurotoxicity, addiction, and other side effects, significantly limiting its clinical application. Agomepratine has no significant improvement in the treatment of depression in over one-third of patients (Perrine et al., 2014; Lorman, 2019). At the same time, although chemicals regulating endocrine metabololism such as metformin have been proven to have an antidepressant effect in preclinical and clinical studies. However, there is a risk of increasing the incidence rate of cardiovascular disease, and it will also cause adverse reactions of digestive system symptoms such as diarrhea and indigestion (Xiao and Luo, 2018).

TCM can regulate autophagy through multiple compounds, targets, and pathways and has great potential in the treatment of MDD. However, most of the current studies on TCM still require further validation through clinical experiments. Many active compounds in TCM have limitations, including poor stability, poor solubility, and difficulty crossing the blood-brain barrier. Additionally, the specific targets of autophagy-related genes in TCM need to be further clarified through mechanistic studies. More importantly, this review highlighted inconsistent findings regarding the inhibition or enhancement of neuronal autophagy, suggesting that the influence of neuronal functional activity during the treatment of depression cannot be disregarded.

Therefore, future research should focus on conducting clinical observations to assess the therapeutic effects and adverse reactions of TCM in MDD patients, as well as investigating the regulatory effect of autophagy in these patients. Moreover, efforts should be made to develop targeted delivery systems for TCM to enhance drug concentration and duration of action in the central nervous system, consequently improving the therapeutic effect of TCM on target organs. Combining multi-omics technology with these studies would further enhance our understanding of the mechanisms and functions of TCM in autophagy regulation, improve our understanding of the pathological mechanisms of autophagy-induced depression, and elucidate the specific roles of neurons and their relationship with autophagy.

## 6 Conclusion

To sum up, autophagy is closely related to the pathological mechanism of MDD. This review further explores the upstream and downstream molecular mechanisms of autophagy affecting MDD, summarizes the relationship between autophagy and MDD related molecular signaling pathways, and further analyzes the pharmacological mechanisms of antidepressants on this basis, in order to provide new strategies for the treatment of MDD patients. However, in both clinical and preclinical studies, more research is needed to explore the mechanisms underlying autophagy regulation by antidepressant agents, which is of great significance for the research and development of TCM in the field of depression therapeutics.

## Figures and Tables

**Table:** 

**TCM**	Traditional Chinese medicine
**MDD**	Major depressive disorder
**SSRIs**	Selective serotonin reuptake inhibitors
**5-HT**	Serotonin
**CMA**	Chaperone-mediated autophag
**mTORC1**	mTOR complex 1
**ULK1**	Unc51 like autophagy activating kinase 1
**PI**	Phosphatidylinositol
**PI3P**	Phosphatidylinositol trisphosphate
**PI3K**	Type III Phosphoinositide 3-kinase
**SNARE**	Soluble NSF attachment protein receptor
**NLRP3**	NOD-like receptor pyrin domain containing 3
**IL-1β**	Interleukin-1β
**HMGB1**	High molecular group box 1
**STAT3**	Signal transducer and activator of transcription 3
**NF-κB**	Nuclear factor-kappa B
**mPFC**	Medial prefrontal cortex
**NSCs**	Neural stem cells
**NPCs**	Neural progenitor cells
**SVZ**	Subventricular area
**SGZ**	Subgranular area
**DG**	Dentate gyrus
**CORT**	Corticosterone
**ATG**	Autophagy-related gene
**BDNF**	Brain-derived neurotrophic factor
**MMP9**	Matrix Metalloproteinase 9
**AMPK**	Adenylate-activated protein kinase
**DA**	Dopamine
**NE**	Norepinephrine
**E**	Epinephrine
**Nrf2**	Nuclear factor (erythroid derived 2)-like 2
**LPS**	Lipopolysaccharide
**PPD**	*Postartum* depression
**Gαi-2**	G alphai (2)
**PKA**	Protein kinase A
**ASK1**	Apoptosis signal-regulating kinase 1
**NMDAR**	N-methyl-D-aspartate receptor
**RAGE**	advanced glycation end products
**CUMS**	Chronic unexpected mild stress
**TZD**	Thiazolidinedione
**H2S**	Hydrogen sulfide
**SIRT1**	Silent mating-type information regulation 2 homolog 1
**TrkB**	Brain-derived neurotrophic factor-tropomyosin-related kinase B
**PDE4**	Phosphodiesterase-4
**COPD**	Chronic obstructive pulmonary disease
**DHA**	Docosahexaenoic acid
**FOXO**	Forkhead box o
**VE**	Vitamin E
**NEK7**	NIMA-related kinase 7
**AMPARs**	α-amino-3-hydroxy-5-methyl-4-isoxazole propionic acid)-type glutamate receptor
**NIX**	Nip-like protein
**PGC**	Peroxisome proliferator-activated receptor-gamma coactivator
**Mfn2**	Mitofusion 2
**ER**	Endoplasmic reticulum
**mtROS**	mitochondrial reactive oxygen species
**Cx43**	Connexin 43
**XYS**	Xiaoyaosan
**GLUT4**	Glucose transporter-4
**TSPO**	18 kDa translocator protein
**AKT**	Adenylate-activated protein kinase
